# Building Dynamic Communities of Interest for Internet of Things in Smart Cities

**DOI:** 10.3390/s20102986

**Published:** 2020-05-25

**Authors:** Monira N. Aldelaimi, M. Anwar Hossain, Mohammed F. Alhamid

**Affiliations:** Department of Software Engineering, College of Computer and Information Sciences, P.O. Box: 51178, Riyadh 11543, Saudi Arabia; maldelaimi@ksu.edu.sa (M.N.A.); mohalhamid@KSU.EDU.SA (M.F.A.)

**Keywords:** internet of things, community of interest, object relationships, social internet of things, social objects, smart cities

## Abstract

The Internet of things (IoT) is a growing area of research in the context of smart cities. It links a city’s physical objects that are equipped with embedded sensing, communicating, and computing technology. These objects possess the capability to connect and share data with minimal human intervention, which creates the potential to establish social relationships among them. However, it is challenging for an object to discover, communicate, and collaborate dynamically with other objects, such as social entities, and provide services to humans. This is due to the increase in the number of objects and the complexity in defining social-like relationships among them. The current research aims to address this by introducing an object architecture and defining a Dynamic Community of Interest Model (DCIM) for IoT objects. The proposed model will help IoT objects to socialize and build communities amongst themselves based on different criteria. In this approach, objects belonging to a community will collaborate with each other to collect, manipulate, and share interesting content and provide services to enhance the quality of human interactions in smart cities.

## 1. Introduction

Due to the emergence of the Internet of Things (IoT), the idea that physical things could communicate with each other and provide humans with services, without intervention, has evolved from imagination to reality in recent years. In IoT, physical objects have the capability to communicate with each other without any or with minimal human intervention. These objects can be any devices, appliances, vehicles, buildings, or any other physical objects equipped with embedded sensors, software, and network connectivity to provide services for citizens in smart cities [1,2].

However, it is challenging for an object, without intervention, to discover other emerging objects in its environment, to form social connections for interacting and collaborating with surrounding objects, and to provide required services to individuals around it in the physical world. The challenge lies in the fact that there has been a significant increase in the number of IoT devices [3], which has introduced complexities in defining the variable social relationships among these heterogeneous objects. The current research aims to address this issue by introducing a community-based model for IoT objects aligning to the concept of the Social Internet of Thing (SIoT) network [4,5,6].

Relationships among IoT objects have been discussed in previous literature [5,7] from various perspectives. These relationships are exploited to form IoT objects’ communities or clusters based on different criteria, such as interest or location. It has been observed that many of the existing works propose static clustering of objects based on a centralized approach, whereas the current research proposes a decentralized and dynamic approach to form communities of interest.

The proposed model, named the Dynamic Community of Interest Model (DCIM), aims to group IoT objects to form a community of interest where devices belonging to one community can easily communicate and share relevant content with other devices in a dynamic fashion. Sharing services among only interested objects in a community leads to better efficiency, fast and accurate fetching of results, time saving, and more effective interaction with people [8]. Furthermore, the dynamicity of the communities is advantageous, since many of the devices are mobile in nature, such as smart phones and vehicles. To form such communities of interest and build a successful relationship between IoT objects, devices’ behavior and relationship factors need to be analyzed.

The contributions made in this paper are highlighted as:

- Analysis of the properties and capabilities of IoT objects in a smart city context and investigation of several types of relationships among those objects to support building communities.
- Proposal of a model to form dynamic communities among discovered IoT objects based on the defined interests and new relationships among those objects.
- Simulation of the proposed model using a real-world scenario and evaluation of its effectiveness.

The remainder of the article is organized as follows. Section 2 reviews related work and Section 3 provides analysis of community aspects in IoT along with the relationships. Section 4 proposes the SIoT architecture and the DCIM model. Section 5 shows the utilization of a simulation tool to demonstrate the model and evaluates the different aspects of the model, and finally Section 6 concludes the article with future work directions.

## 2. Related Work

Recently, we have noticed a flourish in proposals intended to transform IoT smart objects into social IoT objects by giving them social-like capabilities. This section summarizes a broad spectrum of works that are related to the topic.

### 2.1. Relationships in IoT Objects

The concept of object relationships in the IoT has been discussed in the literature. Kosmatos [7] explored social network-like relationships and outlined existing IoT architectural models, from RFID, smart object, and social perspectives. Objects in this architecture can participate in communities of objects, form groups of interest, and collaborate. However, the mechanisms to build the desired social networks of objects are not discussed in this paper. Researchers in [9] consider the relationships between IoT objects by creating Social Virtual Objects (SVOs) that correspond to real world objects (RWOs) in an edge-cloud environment. Although the SIoT paradigm is discussed with a focus to zone-based partitioning of SVOs, the authors do not address the dynamic community formation aspect among the IoT objects.

Friendship selection in the SIoT is introduced in [10], which addresses the problem of overpopulated objects in the IoT by introducing link selection strategies to improve network navigability. This work of friendship selection and network navigability can be leveraged further for dynamic grouping of IoT objects for collaboration.

### 2.2. Social Internet of Things

The SIoT paradigm has appeared recently in many studies [4,5,11,12,13]. In some of the early research [3,5], the authors identified policies to establish and manage objects’ social relationships. To characterize the SIoT network and its navigability, authors studied the probability distributions of two variables, the geographical distance between connected nodes and the shortest path length between a pair of random nodes. The distance between nodes can work as a measure of proximity, which can be used for dynamic community formation.

SIoT is introduced in [11] in a distributed environment, were the objects can search for a service using its relationships and friendships. However, the search can be inefficient when an object has to manage a large number of friends. The researchers addressed this issue by presenting heuristics-based link selection strategies for the object to find a desired service. In this work, the network behavior is analyzed in terms of average path length, large component, average degree, and local cluster coefficient. Here, social aspects were used to search for services, not for community building.

Researchers have identified similarities between SIoT features and social networks of humans [12]. This research identified several key aspects of SIoT that include social roles, intelligence, socialized devices, and everything as a service. Moreover, Roberto Girau [13] proposed an experimental platform for SIoT where objects are able to create their own relationships and groups according to the set of rules as determined by their owners. Here, the owners determine objects’ characteristics such as name, description, mobility, and relationships they possess, including ownership object relationships (OORs) or social object relationships (SORs). The relationship of an object with others can play a vital role in the formation of dynamic communities between them.

### 2.3. Mobility Awareness in Social Relationship

Mobility-awareness of IoT is taken into consideration in [14] where social relationship attributes of mobile nodes, community property, and activity rules of nodes are analyzed to improve the discovery efficiency of nodes and increase the success ratio of the awareness service. They proposed a social relationships cognition algorithm, which extracts interconnection and distance factors to easily construct and quantize a social relationship between nodes.

Authors in [15] introduced information, objects, and people as the three major elements in human society to build relationships using the Internet, the IoT, and the social network, respectively. In the mobile wireless sensor network domain, the authors in [16] proposed a dynamic clustering approach to cluster mobile sensor nodes, which can be utilized in a mobile IoT context. In general, the mobility awareness aspect helps in building a dynamic social internet of things, which is the goal of many emerging studies.

### 2.4. Social Internet of Vehicles

The Social Internet of Vehicles (SIoV) has been discussed widely in many recent papers. Some studies apply a social object concept to the platforms for managing vehicles [17,18,19,20]. The SIoV paradigm introduced in [17] is an extension of the SIoT concept proposed in [5]. In this paper, researchers integrated vehicular ad-hoc networks (VANETs) into the SIoT to conceptualize the SIoV. Mobile nodes in VANETs are the vehicles and static nodes are the road-side units (RSUs). The social relationships between the vehicles and between the vehicles and RSUs can be further analyzed to investigate the formation of the dynamic community in this domain.

Similarly, Kazi M.A [18] utilized the available cloud-based VANETs, such as vehicle-to-vehicle, vehicle-to-infrastructure, and vehicle-to-internet communications. This research defined the SIoV as a vehicular instance of SIoT and studied different forms of relationships, which can be leveraged to form dynamic or static friendships with other SIoV components.

### 2.5. Community of Interest for IoT

Communities that result from objects’ relationships for IoT are discussed in [21,22,23,24]. A community-based architecture for IoT is proposed in [21], which is based on information-centric networking (ICN), called Data-Clouds, to better accommodate data-centric services. The community formation takes into account the sharing nature of data-centric services in the IoT, where the users subscribe to services based on their interests. Various communities in the network are formed by grouping users with common interests together.

Authors in [22] proposed a community detection scheme in the SIoT (CDIISN) using a graph mining approach, where nodes and actors in the complex network are divided into basic nodes and IoT nodes. Any two nodes are considered a community if there exists only one hop distance between them, and they have no less than two mutual friends. Furthermore, a node can be a member of multiple communities. The CDIISN algorithm uses mutual friends as a metric for extracting communities. Negatively, however, this work is not generalized to all networks, such as directed networks.

The idea that social devices are inherent to the community is discussed in [23], which focuses on enriching local interactions in a mobile cloud domain. Their main focus was on autonomous interaction that occurs between mobile phones. Here, the devices’ relationships depend on the limited actions specified by the developer of the platform rather than on the interest.

Likewise, L. Atzori [24] analyzed the major opportunities resulting from the integration of social networking concepts into the IoT. This research presents a social object concept to share a common interest to communicate and enhance the trust between objects. In comparison to the above works, the current work envisions a network based on relationships among objects that can provide useful services on-the-fly for humans in a smart city, and help social objects to communicate within dynamic communities based on common interest.

In summary, to show the main differences between our approach (DCIM) and other approaches found in the literature, a comparison is provided in Table 1, which justifies the need for the proposed work. The comparison is based on the following:

Object relationships—Refers to the types of IoT objects’ relationships. In Table 1, “General” means that the proposed work talked about the relationships in general without specifying any type.

IoT devices considered—The IoT devices that participate in the relationships. Some works deal with RFIDs or vehicles, while some others are scalable for different devices.

Community Formation—This criterion shows if the proposed work supports the formation of communities among IoT objects.

Architecture Proposed—The different IoT architectures proposed by researchers.

Architectural distribution—Refers to the type of architecture solution proposed, whether centralized or decentralized.

Object relationships Implementation Status—Illustrates whether the research goes through the implementation phase for forming the objects’ relationships.

Table 1 uses several acronyms for object relationships, which are POR: Parental Object Relationship; CLOR: Co-Location Object Relationship; CWOR: Co-Work Object Relationship; OOR: Ownership Object Relationship; SOR: Social Object Relationship; GOR: Guardian Object Relationship.

## 3. Analysis of Community Aspects for IoT

IoT objects are ubiquitous, but they are not currently utilized to form relationships and communities for effective collaboration. This section explains social concepts in the IoT, analyzes social object capabilities, discusses IoT object dynamicity, and categorizes the different relationships that can be possessed by IoT objects in order to mimic social behavior. Finally, it defines a new relationship that is incorporated into the proposed model.

### 3.1. Social Concept in IoT

If used in silos, IoT objects have limited capabilities and are narrow in scope, thus providing less benefit to people. These limitations can be overcome by giving the IoT object a social characteristic, which is the focus of this research. The application of a social concept in the IoT allows people and technology-embedded objects to socialize and interact within a new social framework that would be promising for smart cities [2]. This would enable a variety of attractive applications where the objects can participate actively with different social roles. The resulting network is thus called the SIoT [4,6,25].

### 3.2. Social Objects’ Capability

The social object is an important aspect in our approach. It is an entity with social properties, and can be anything, either human, devices, or physical objects embedded with technology, such as mobile phones, vehicles, and buildings. According to [24], social objects have the capability to (1) autonomously interact with other objects, join/leave different communities, and shape their own social network; (2) discover services and information by crawling the IoT systems which are made of millions of objects; and (3) provide services to other objects by advertising their presence. The capabilities of social objects affect the structure of SIoT networks by bringing up new forms of relationships. In this research, the proposed model incorporates social objects that possess the above-mentioned capabilities.

### 3.3. Social Objects’ Dynamicity

IoT systems consist of millions of heterogeneous devices that can be either static (e.g., smart-board) or dynamic (e.g., vehicle) depending on their mobility. The dynamicity of a social object does not only depend on its mobility [23], but also on the change of object status. Examples of status change may include (1) devices can be turned to silent mode by their owner or by encountering a specific situation; (2) IoT devices may run out of battery; and (3) the object profile may change over time based on the updates done by the owner, such as updating owner’s rules or engagement or interests. This research considers the dynamic aspects of social objects while proposing a community of interest model for IoT objects.

### 3.4. Social Objects’ Relationships

Social objects can be bound by different kinds of relationships among them. Social objects participating in the SIoT can collaborate and share data utilizing their relationships. There are many factors that affect the creation of a relationship [26], such as object type, computational power, mobility, capability, brand, and frequency in meeting the other objects.

**Figure 1:**
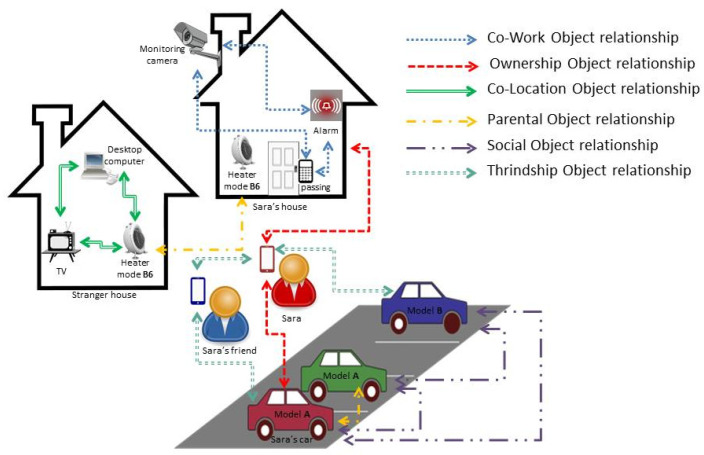
SIoT objects relationships comparison.

In the literature, there are many representative studies [4,12,27] that discuss different types of relationships possessed by IoT objects. They promoted the idea that objects and human stakeholders must be considered in the SIoT objects’ relationships. In this section, a summary of these relationship types, as well as an example of each, is presented. Figure 1 helps to understand the differences between these relationships.

#### 3.4.1. Parental Object Relationship (POR)

This type of relationship is formed between similar objects that have been produced in the same production batch; this means that the objects in this relationship are homogeneous objects originating in the same time period and with the same manufacturer. As the relationship is formed during item production, and will not change over time, it is a static relationship.

**Example**: Cars from model Mercedes produced in 2010 will, by default, share a parental object relationship; Samsung air conditioner model B2 produced in 1999.

#### 3.4.2. Co-Location Object Relationship (C-LOR)

This type of relationship is formed between objects that constantly exist in the same place. These objects are either homogeneous or heterogeneous objects used always in the same location. This relationship is established as part of the initialization/implementation of a “location-based application” profile. Thus, it is static as long as the implementer decides to change based on the duration of the co-location relationship.

**Example**: Printers, faxes, computers, office furniture, counters, etc., located in the same building have a Co-Location Object Relationship, as do sensors, actuators, televisions, windows, and augmented objects used in a smart home.

#### 3.4.3. Co-Work Object Relationship (C-WOR)

This type of relationship is formed between homogeneous or heterogeneous objects that periodically collaborate to provide a common IoT application. This relationship is established as part of the initialization/implementation of a “situation-based application” profile. Thus, it is static as long as the implementer decides to change based on the duration of co-working, frequency of the interaction, and reputation.

**Example**: Objects that used together and cooperate to perform a specific task. such as objects in security systems.

#### 3.4.4. Ownership Object Relationship (OOR)

This type of relationship is formed between objects that are owned by the same user. These objects are heterogeneous and associated by a common owner. A richer device profile helps to build this kind of relationship.

**Example**: Mobile phones, tablets, and music players owned by Sara, or printers, computers, and any other objects owned by King Saud University.

#### 3.4.5. Social Object Relationship (SOR)

This type of relationship is formed between heterogeneous objects that sporadically or continuously come into contact with each other because their owners come in contact with each other or by their own if they have the ability to move independently of their owners. Objects in this relationship can autonomously share their social profile if they have the authority from their owners.

**Example**: Devices such as phones and tablets that come into contact because their owners are classmates or travel companions, different cars that share a common problem in a specific road can share a SOR. Houses can also share a SOR by sharing data and services.

#### 3.4.6. Thriendship Object Relationship (TOR)

This type of relationship is presented in [28]; it is formed between heterogeneous objects that belong to friends. TORs can be described as the friendship among things/devices of friends; each object is associated directly with its owner friendships.

**Example**: The mobile phone of Sara and tablet of Sara’s friend will have a TOR; the television of Sara with the television or any device belonging to Sara’s friend.

#### 3.4.7. Common Interest Relationship (CIR)

In addition to the different relationships found in the literature, we introduce a new kind of relationship among social objects in the SIoT scenarios. This is termed a common interest relationship (CIR), which is triggered by geographical proximity of the objects subject to having common interest among them. This is unlike the SOR that only depends on the location. Objects in CIR-type relationships need to be authorized by their owners and also have to check their owners’ rules before each contact. Similar to the SOR, the objects in the CIR are heterogeneous and the relationship is non-static.

**Figure 2:**
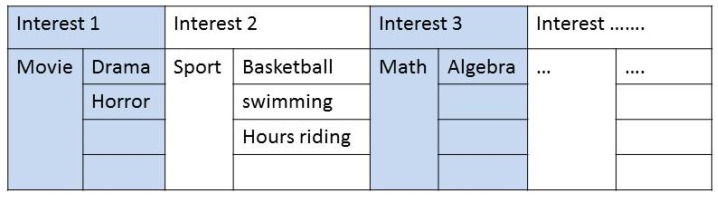
Interest list structure.

Modeling a structure for common interests is essential for handling and manipulating the interests from different IoT objects. The structure used herein is a kind of list structure with length depending on how many key-interests there are. Each element in the list is composed of a key-interest and value where each interest may have one or more values. These values can be entered by the device owner to describe his/her interest. An illustration of this structure is shown in Figure 2.

**Figure 3:**
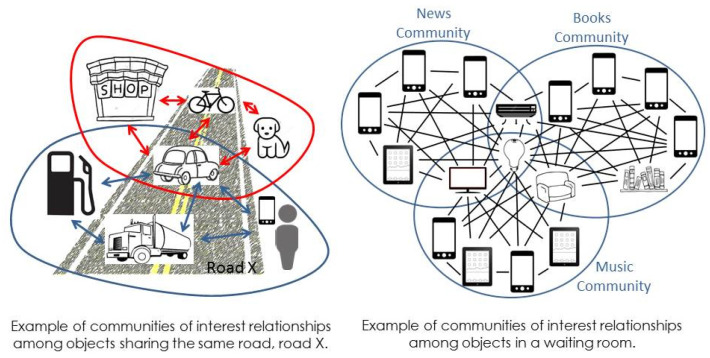
Examples of common interest relationship (CIR).

**Example**: Devices people own and are with them in the same waiting area can create and join CIRs among each other and with any objects there, such as TVs, ACs, book shelves, or seats. They can communicate, share their interests, and enjoy waiting. As a further example, vehicles, shops, people, and any objects that share the same road at a specific time, which can recognize each other, share a common problem, and enjoy sharing videos, music, or any kind of information, belong to the same community of interest, in this case the community of road X. Figure 3 illustrates these two examples.

## 4. Proposed System

In order to provide the detail of the proposed dynamic community of interest model, we first analyze the existing social IoT architecture and accordingly propose a distributed social IoT architecture for establishing coordination between IoT devices. This is followed by the detail of the DCIM model.

### 4.1. SIoT Architecture

**Figure 4:**
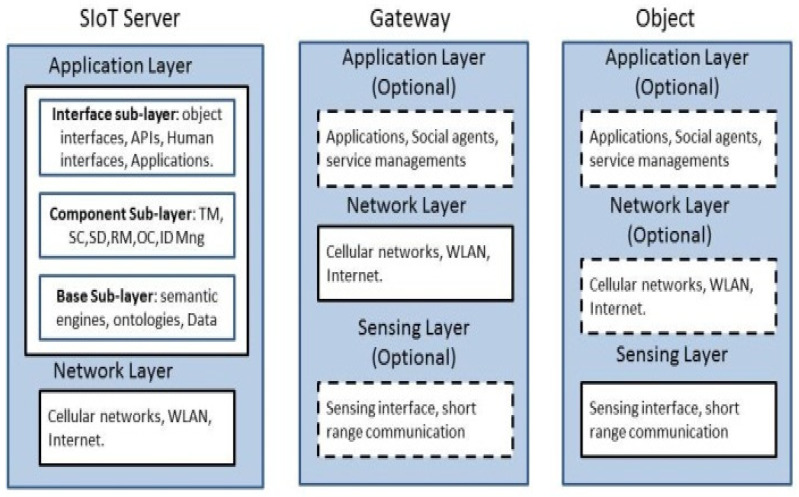
SIoT Architecture.

There is no unified agreed architecture in the literature for SIoT systems due to the novelty of the concept and the variety of its application domains. The current state-of-the-art IoT architectures are discussed in [29] from different perspectives. One of the proposed system architectures [30] is a high-level architecture followed a three-layer model, which consists of the Sensing Layer, Network Layer, and Application Layer, as illustrated in Figure 4. These layers are distributed in three basic architectural elements, which are the SIoT Server, the Gateway, and the Object. These are described in as follows.

**SIoT Server**: As illustrated in Figure 4, the server consists of the Network and Application layers. The Application layer has three sub-layers: (1) the Base Sub-layer includes the database for the storage, handles the management of the data, and defines relevant descriptors; (2) the Component Sub-layer includes the tools that implement the essential functionality of the SIoT system as proposed in [27]; (3) the Interface Sub-layer provides interfaces to objects, humans, and services.

**Gateway and Objects**: in the Gateway and Objects elements, the three layers are not static. In the Gateway, the Sensing layer and the Application layer are optional, whereas in the Objects the Network layer and the Application layer are optional. These combinations of layers may vary from one system to another depending on the device characteristics, which is illustrated in [27] using different scenarios.

Another high-level SIoT architecture is proposed in [12] that has four main elements: Actors—such as smart things and users; Intelligent System—to manage and orchestrate all the interactions undertaken by the actors, in addition to handling Service and Applications Management, Recommendation, Service Discovery, and Data Management; Interface—for actors to enable the interactions between them, such as the input of data and queries, as well as the requested output; Internet—to provide open access to all the involved entities, and acts as a communication medium for bringing smart devices with their services to the users and allows them to interact with their devices and services.

### 4.2. Proposed Architecture

**Figure 5:**
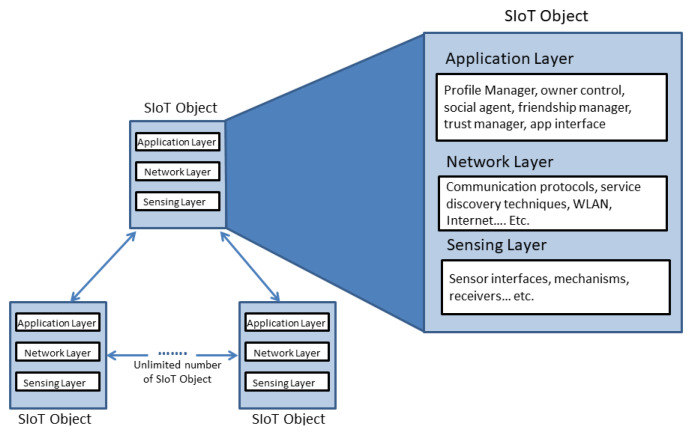
Proposed architecture.

Based on our analysis of general SIoT architectures, we found that many of the community formation studies follow a centralized approach, which suffers from a scalability issue. This is because the workload of the central server unit is affected directly by the number of connected devices [31]. In our approach, the management components or the architectural elements are located in the object itself and each object builds its own relationships to navigate the network, thereby following a distributed architectural approach, as suggested in [6]. Each object will have its own management parts organized in three layers, namely Sensing layer, Network layer, and Application layer, as proposed in [27]. Each of these layers consists of several modules. This solution will enable effective collaboration in the relationship management functionality between all objects. Objects will have the capability to interact with other objects and share data directly without a need for a central server. The proposed architecture is illustrated in Figure 5 and is described below.

**The Sensing Layer**: The core function of this Sensing layer is to gather data from the surrounding environment, such as temperature, light, object emergence, distances between objects, weight, or any data sought by the system through sensor interfaces. These data can be collected through embedded technologies in the object, such as micro-sensor cells, RFIDs, readers, and receivers. Some objects also may gather data by other mechanisms.

**The Network Layer**: The Network layer is responsible for detecting other objects and discovering desired services in addition to the management of communication protocols. The object unique identity in the social network of things is identified and processed in this layer.

**The Application Layer**: This is the main layer in the architecture, where it is responsible for managing the object relationships, communication rules, owner settings, and information processing. In this layer, we find the Profile Manager, to manage the device’s basic information and interest records of devices’ owners; Owner Control, to enable the object owner to specify his/her interaction rules and to apply and manage these rules; and Social Agent, which is responsible for enabling the object to socialize, communicate with other objects, and start, update, and terminate their social relationships. Finally, the Friendship Manager, which is responsible for identifying objects’ friends, i.e., those who were previously communicated with and built relationships. Furthermore, this layer takes the role of protecting the object from any malicious contact through a Trust Manager module.

### 4.3. Dynamic Community of Interest Model

Within the context of the architecture defined in Figure 5, we propose the DCIM model, which exploits the social characteristics of IoT objects, their capabilities, and the CIR relationship. DCIM proposes a social network-like group formation approach for IoT objects. This enables objects in IoT systems to mimic human behaviors that are exposed in their real lives. In this model, the formation of the community of interest, and joining and leaving these communities, are the responsibility of the objects themselves with minimal human intervention. The objects’ owners are responsible only at the beginning stage for defining his/her own interaction rules and interests. Objects participating in this model can be phones, devices, sensors, appliances, vehicles, buildings, animals, or any other “physical things” with embedded communication and computing technology. The proposed model assumes each object has a unique identity that can be easily identified and interacted with.

#### 4.3.1. Characteristics of Prospective Community

One of the characteristics of the proposed community of interest is its dynamicity, which comes from the mobility of IoT objects. Thus, the community can be formed among objects while they are moving. The number of communities is not static; it can be formed whenever two objects or more satisfy CIR conditions, and disappear whenever these conditions are not satisfied. Each community built using a DCIM has one common interest, but objects with several interests are allowed to join several communities. Thus, it is possible to have an overlapping between two or more communities when multiple objects join these communities at the same time. Objects in one community have a high density of interaction and strong relationships unlike other objects from different communities.

#### 4.3.2. Criteria Used to form Community of Interest

The different criteria used to form the dynamic community of interest with the IoT objects are the common interest between these objects, interaction rules defined by the object’s owner, and the communication range of these objects. These factors are described as follows.

- Common Interests

Building dynamic communities of interest for the IoT mainly depends on the common interest between social objects, which is described in Section 3.4.7. Thus, in order for an object to pursue common interests with others, it keeps a reference to its own interests in the device profile. These interests are determined by its owner at the initial stages of object setup that can be managed and updated through the Profile Manager module of the proposed architecture given in Section 4.2. The device’s profile is open for the public to be checked by all nearby objects since it is the main criteria in this proposed model. The owner’s interest or device’s profile can be anything, such as sports, art, politics, a common goal, or a common trip. For enriching the DCIM, similar terms will be detected and analyzed to link the objects with a common interest but different expression together. As an example, if two objects are interested in Cartoon and Animation, respectively, then they are actually sharing the same interest; thus, the DCIM will detect this similarity and build a common interest relationship with them. Furthermore, the owner can prioritize his/her owner interests in case, for example, there are two available communities to join and the owner’s rules don’t allow joining of more than one community.

- Communication Range

In this model, to start any interaction between two objects, they need to be within each other’s communication range. This means that the communication range between different devices is a pre-condition for the creation of the common interest relationship between those objects. The range for each object that allows it to detect neighboring objects is identified by the techniques or protocols followed by the objects and provided by the Network layer.

- Social Interaction Rules

In addition to the interests, each object has its own private social interaction rules decided by its owner at the beginning of establishing the object using the owner control component. These interaction rules can be considered as the unit that enables the object’s social activities, and identifies the information that can be shared and the type and number of relationships allowed for an object to join. Thus, interaction rules help to manage an object’s relationships and reduce owner intervention while interacting, sharing, and forming the communities of interest. We consider a key-value style structure to define these rules. Example includes:

○ Number of objects in a community that a new object is allowed to join.
○ Type of objects in the community.
○ Duration allowed for an object to be in the community.
○ Number of communities that an object can join at the same time.

It should be noted that there is a Trust Manager module to handle malicious contact in the current architecture. However, the DCIM does not specifically consider security and privacy factors as a criterion for community building. Future versions can incorporate these factors to make the model more robust against malicious IoT nodes willing to join a community.

#### 4.3.3. The Proposed Model Description

The DCIM model can be elaborated further via the following steps.

- First, a dynamic object emerges; it is identified by all existing objects and vice versa.
- Interests’ records or devices’ profiles are shared among them to find common interests.
- If there is a community in the communication range of the new object that shares an interest with the newly emerged object, the object will join the community and all existing objects in the community will be aware of this new object.
- If there is no community that matches the interest of the new object, the new object will try to find other objects in the vicinity that have a common interest. If a common interest is found between any two objects, they will send a community formation request to each other. If the request is accepted by both objects, the community will be formed and ready to welcome any other objects that emerge.
- In this model, an object can leave a community at any time based on its defined interaction rule, or when it moves out of the communication range.
- The minimum number of objects in a community is two, however, there is no limitation on the number of objects that can be joined in the community.

### 4.4. Applicable Scenarios

The DCIM can be applied in many application areas in order to provide services and facilitate living. This model also can be used to improve existing smart systems and integrate them to be social systems, such as smart homes, smart cities, and universities. We illustrate here some applicable scenarios that utilized our DCIM.

#### 4.4.1. Smart City Airport Scenario

In an airport, as a motivated scenario, travelers check information boards or counters to gather information regarding their trip, such as time and location. They also need to concentrate on all audio announcements in order to catch their flights. Furthermore, waiting in airports for long periods is not convenient for most people. With DCIM, all of these issues can be addressed by creating different kinds of groups of interests (communities) between IoT objects in an airport. As an example of some communities, all mobiles or devices of people who are taking the same flight will be in one community at the airport, the information center, flight gate, café, plane, etc. Any trip information, announcements, and changes of plan can be shared among only people who are interested in this trip. Information can reach the right persons by notifications via their phones or tablets. In addition, people who search for specific information regarding the flight can post a question, while others having specific information regarding the flight can easily post their information to the trip community. Thus, wasting of time due to checking information counters and listening to announcements, and concerns regarding changes in flights details, are eliminated. Furthermore, people in the airport can join a community of their interest, such as sport. Hence, they can take advantage of the waiting time by sharing information of interest and discussing their common interest. Book shelves and items in duty free stores can join these kinds of communities.

#### 4.4.2. Social Communities in Smart University

This reflects building different communities in the context of smart city universities for effective collaboration; for example, building a community of devices belonging to the same course offered in a department. When the class (a class is also considered as an IoT object, which embeds other sensors and technologies) detects students’ devices registered for course A (course A as a community), at the time of course A lecture material will be downloaded by those devices automatically due to the trusted relationship established between devices in the community. In addition, when the class sensor detects a faculty member who teaches course A, lecture material will be presented directly on the screen by the class projector. Attendance will be recorded automatically by the class sensor that can identify students’ devices. Furthermore, when the class detects a device that does not belong to current course community, an alert will be sent to the faculty to obtain her/his permission.

In addition to the communities of courses, different groups of interest can be created dynamically with the proposed DCIM model among the devices of students, faculties, and staff while they are in the vicinity of any IoT objects, such as printers, ACs, or library book shelves. Thus, they can share common interests and services. As an example, if a student is interested in mathematics and he is passing the library with his mobile, a community of mathematics may be formed with other devices that reflect the shared interest of others. In this community, the library itself can post any information about new books that have arrived, or student devices can seek relevant information that was searched for by the student. Various other forms of the smart university campus can exist, such as that in [32], which can benefit from the DCIM model.

#### 4.4.3. Connected Vehicles

The concept of connected vehicles is a vision in which communication and interaction in the form of vehicle-to-on-board sensors (V2S), vehicle-to-road infrastructure (V2R), vehicle-to-vehicle (V2V), and vehicle-to-Internet (V2I) is enabled [20]. The IoT plays an important role in realizing this vison because it is one of the building blocks. The primary goal is to offer vehicle and driver assistance, share information, provide infotainment, and provide other services to enhance the experience of passengers and improve the overall transportation system.

The proposed DCIM model can be applied in the connected vehicle domain, where vehicles that are close to each other can interact for different purposes. For example, when a vehicle faces some problems or needs any assistance, it may send a message to all vehicles in the proximity or to those passing on the same road at that time. The vehicles can thereby form dynamic communities of vehicles to collaborate and provide assistance. In another example, mobile vehicles can join communities of different interests to share common content.

## 5. Simulation and Evaluation

The proposed DCIM model was evaluated using simulation and the modeling technique of Agent-Based Modeling (ABM). This section presents the simulation framework environment describing the use of ABM with the NetLogo tool [33]. In addition, the airport scenario provided in Section 4.4.1 is implemented here and described in detail. The discussion of results is found at the end of this section.

### 5.1. Simulation Framework

In order to implement the DCIM and study the social behavior of objects in the IoT, a large number of heterogeneous objects is required, including mobile devices, with different profiles and interests. Since it would be difficult to implement the model with such a large number of objects using the required embedded technologies in reality, we used a simulator framework to observe objects’ behavior where an approximation of a real-world scenario can be provided. To simulate the scenario in the NetLogo simulator, ABM was used, which is suitable for modeling social systems containing many agents interacting and influencing each other. Here, the ABM language was used for experimental purposes utilizing the proposed model for building dynamic communities.

#### 5.1.1. Agent-Based Modeling

ABM is a powerful simulation modeling technique that has been increasingly used as a modeling approach in various domains, such as natural, social, and engineered complex systems. The main elements of a system are considered as agents in ABM, where each agent has its own set of attributes that parameterize its operation and behavior to guide the interactions with other agents and the environment [34].

**Figure 6:**
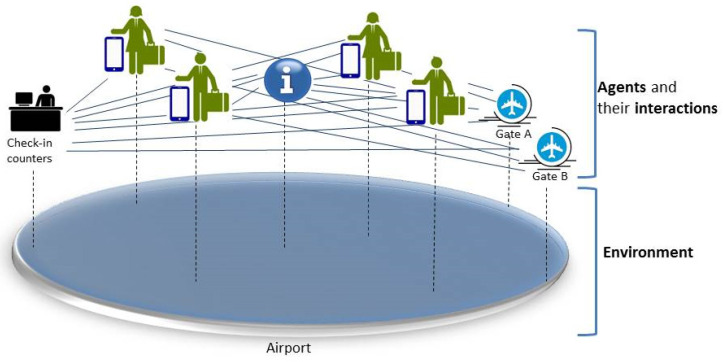
Example of Agent-Based Modeling system structure.

Here, we utilize this technique for accomplishing the main objective of this research, which is to observe the social behavior of IoT objects and build communities of interests among them. In ABM, there are no central management components [35] that manage agents’ behavior and interactions. Rather, ABM systems are decentralized systems where the agents are embedded within devices and provide the communication between each other. The structure of a typical ABM system consists of three main elements [35]: agents, agents’ interactions, and the environment, as shown in Figure 6. These three elements are identified here depending on their usage in modeling the IoT-based scenario.

**Agents**: Each agent in the system represents an IoT device, and has attributes that can be static, such as agent name, or dynamic, such as history of interactions. In addition to attributes, an agent has operations that include agent’s behaviors, such as rules and method of interaction with other agents. Agents have general characteristics from a practical modeling standpoint, as listed in [35], such as social, self-contained, modular, uniquely identifiable, autonomous, and independent. Agents may also be adaptive, goal-directed, and heterogeneous. An agent can be static, such as an information center, and dynamic, such as mobile phone.

**Agent relationships**: A set of guidelines that identify agents’ interactions with each other, the relationships that they can possess, and with whom they can interact. Furthermore, the type and amount of data allowed to be shared and the methodologies of detecting and interacting with other agents are also identified.

**Agent environment**: In addition to agents’ interactions with each other, agents also interact with the environment, which is comprised of anything around the agent depending on the system and their location and movements. For example, in the airport scenario, the environment is the airport with all facilities inside.

The use of agents [33] will help to handle the major issues in the IoT, such as the interoperability between different standards, data formats, interfaces, heterogeneous hardware, protocols, resource types, and software. In DCIM, Rule-Based Modeling, a type of ABM technique, is applied since the objects in this model follow predefined rules.

#### 5.1.2. NetLogo Simulator

To facilitate the modeling work, the NetLogo simulation platform [33], which is a popular modeling tool, was used. The choice of this tool was motivated by the fact that many projects have used it to model their systems, particularly in the case social systems, to represent the complex real-world processes to be observed and investigated, and to test hypotheses on these complex systems.

The NetLogo simulator has been used for modeling and simulating natural social phenomena, where static and mobile agents represent different features of the network. It contains built-in graphical interfaces and experimental visualization tools. Models built in Netlogo have the ability to give instructions to hundreds or thousands of autonomous agents operating in parallel. It runs basically in a single machine, but can be extended to run on cluster of computers. In Netlogo, there are four types of agents: turtles (mobile agents representing IoT objects), patches (static), links (between turtles), and observers (observing the simulated environment).

### 5.2. Experiment

In this section we exploit the airport scenario provided earlier in Section 4.4.1. Further detail is provided here for simulation purposes, which is followed by a discussion of the results.

#### 5.2.1. Scenario Analysis

The airport scenario is analyzed here to justify the applicability of DCIM in the simulated IoT environment. In this simulation, the first type of agent considered comprises mobile tablets and phones carried by travelers, which are represented in the interface by the shape of a human since these devices are always carried by humans. These objects are called turtles in the simulator. The second type of agents are the static facilities in the airport, such as information counters, cafeterias, and offices. The final agent is the observer, which is named the scene.

The interface of the DCIM for the airport consists of the view (scene) and buttons to set and initialize the scene, add travelers, add new types of interest, and extract details of communities. In addition, a slider allows easy identification of the number of new travelers, and monitoring of the communities and the objects joining or leaving these communities.

**Figure 7:**
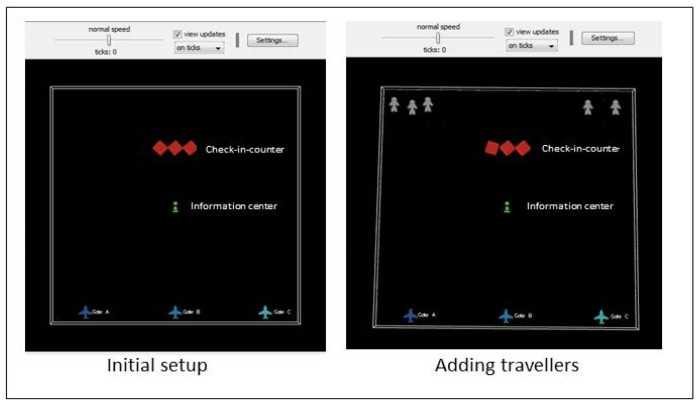
Initial view for the airport scenario.

As an initial step, the view contains three planes behind the airport gates, check in counters, and information centers. Using the “add travelers” and “go” buttons, agents are created and located at the top of the view, which we assume is the location of the airport gate, as can be seen in Figure 7. The travelers move to the check-in-counters where their flight number is identified. Then, travelers randomly move around the airport facilities as in real life. One or more interests are assigned randomly to each object (i.e., the device with the traveler). Since the communication between objects following the DCIM model is influenced by the communication range of objects, each device can interact and join communities only with the devices within its range, which is set as 3 radius units in the simulation tool. Another restriction is the rules set by objects’ owners that specify if an object is allowed to interact and, if not, will deny participation.

In the proposed airport scenario, there are two types of communities: static communities and dynamic communities. The static communities are those communities that formed after travelers have passed through check-in counters and whose flight numbers have been identified. Any two travelers with the same flight number will be in the same community along with the flight itself. This community is static since whenever a traveler joins it, it will not change. The static community is represented in the model interface as a color, where each color represents a different community. The other type is the dynamic community, which depends on the devices’ profiles and travelers’ interests. Whenever a device (owned by a traveler) finds any other device with a common interest, then a CIR will be built between them and there will be a dynamic community of interest. This community is represented in the model interface monitors that provide the number of devices in the community and how this number changes (i.e., increasing or decreasing).

#### 5.2.2. Discussion

The results show the effectiveness of the proposed DCIM to detect common interests among mobile devices and build dynamic communities on the move. The proposed model is evaluated in terms of the number of communities that can be structured using the model (scalability), the number of objects in a community, the overlapping entities between communities, and the dynamicity of the formed communities by observing the object’s behavior in joining and leaving the community.

**Figure 8:**
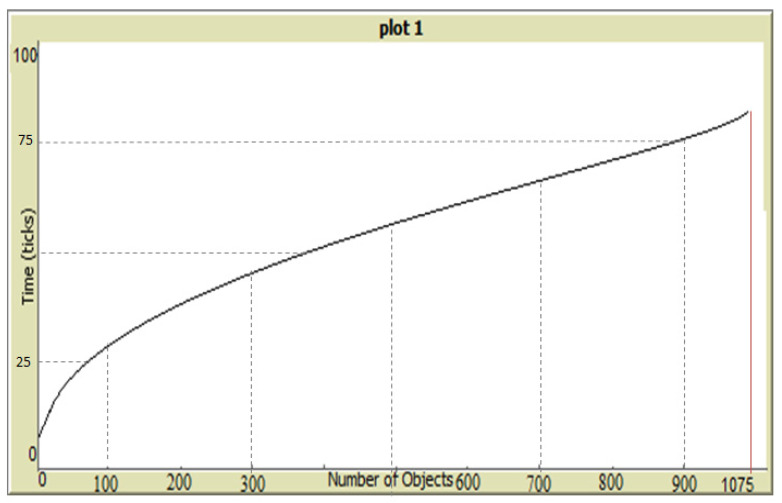
The scalability of the model handling 1000 objects.

In terms of scalability, the DCIM can accommodate a large number of objects that evolve in the social IoT system, and put them in their appropriate community/communities, if they exist, or build a new community if the model criteria are satisfied. Thus, the number of objects in a community is not restricted to a specific number as long as they match the criteria of common interest, communication range, and interaction rules of the objects. This is proved by continually adding travelers in the simulation system, which can be controlled by a slider. Figure 8 depicts a specific case when the simulation handled communities of up to 1000 objects in less than 100 ticks. It is also evident that the number of ticks increases with the increase in the number of objects. However, it can be noted that the rate of increase in the number of ticks is less than the rate of increase in objects, which suggests that the model is scalable, and that each new object handles and manages its own relationships as in peer-to-peer systems within an acceptable time. Here, ticks in NetLogo represent a unit of arbitrary time measurement inside the simulator, which can be adjusted.

The number of communities that can be built using the DCIM depends on the diversity of interests’ types belonging to each device profile. Any new device with a new type of interest that has no community can use the model to search for another device with the same interest and then build a new community. The model can hold as many communities as possible when a new common interest is found. Adding new types of interests is done manually for experimental purposes, using the “adding new interests” feature. During simulation, we started the model with three types of interest, and travelers with new types of interest are then successively added. As a result, the DCIM can be described as a scalable model that allows a large number of objects to interact and form communities, in addition to supporting new types of interests with the emergence of other devices.

**Figure 9:**
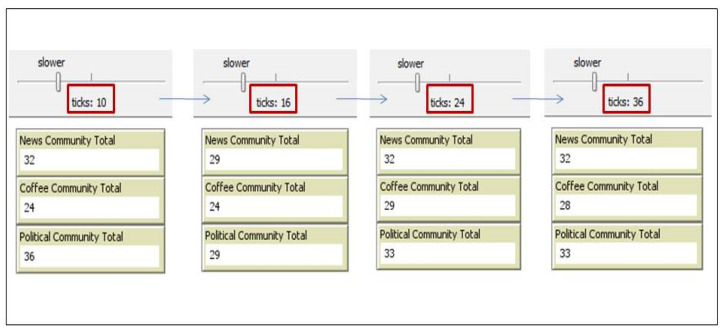
Number of objects in different communities over a period of time.

For testing the dynamicity of the DCIM, we observed how the number of objects changed over time in a community. An increase in the community members means that there is a new object joining the community while a decrease would mean that a member is leaving the community. The community formed by using this model is dynamic in terms of the varying number of community members, unpredictable interest types, the mobility of its members, and the changing object status. To practically show the dynamicity of the model, Figure 9 illustrates the variations in the number of objects in three different communities over time. In this figure, a screenshot is taken after 10, 16, 24, and 36 ticks, respectively. From these screenshots, we can observe that the number of objects in News, Coffee, and Political communities varies.

**Figure 10:**
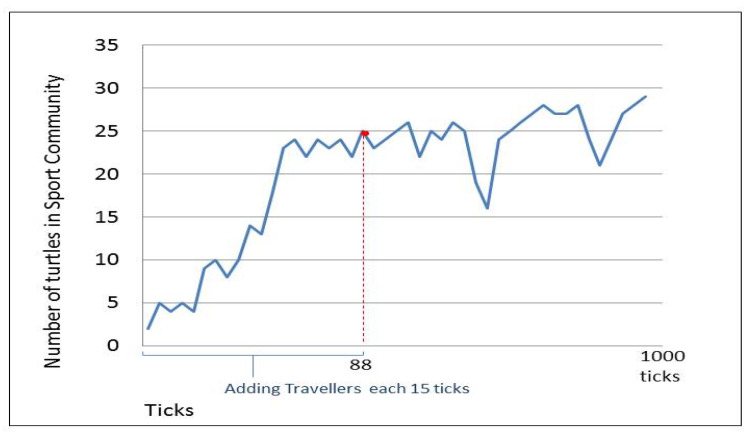
Changes in sport community as an example.

**Figure 11:**
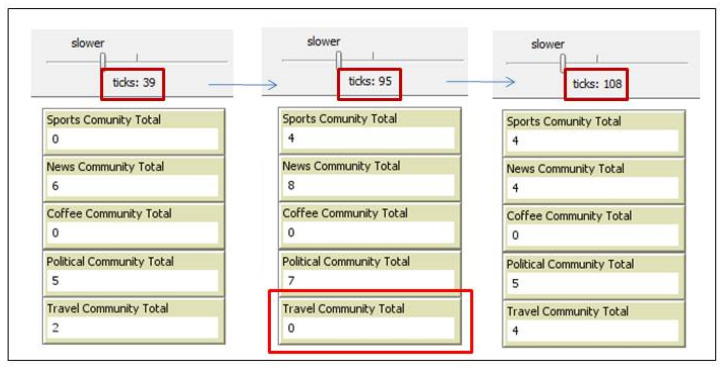
Disappearance of travel community.

Figure 10 focuses on the changes that happen in the community of sport. The figure shows that even after stopping the addition of new travelers at tick 88, the number of devices in the sport community continues to change, since the people holding their devices are moving, causing a change in their communication ranges. We can also observe the disappearance of any community if no member remains in a community, such as the one in the travel community at tick number 95 in Figure 11.

**Figure 12:**
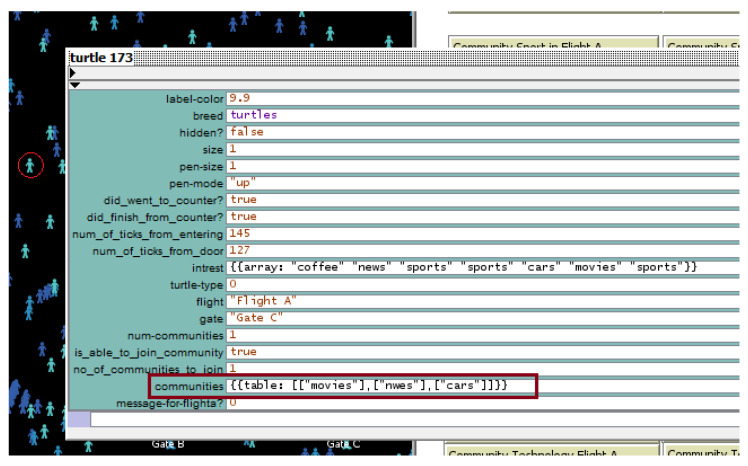
Inspection of object 173.

Since one object can have multiple interests, there is a need to allow overlapping between communities to enable the object to join more than one community of interest at the same time. After simulating the DCIM in NetLogo, we can see that the model allows objects to overlap among different communities. As an example, if we inspect a random object moving in the airport at a certain time, the inspected object behavior in Figure 12 shows that it has seven interests, and at the 127th tick the object joined three communities at the same time, which shows an overlap of these communities.

**Figure 13:**
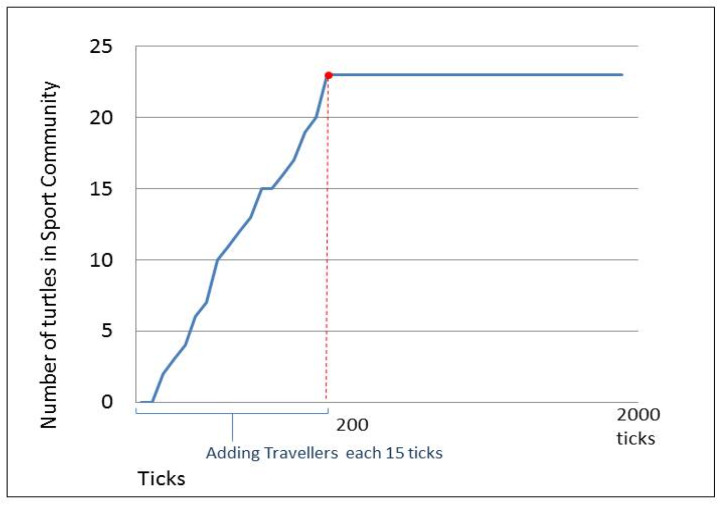
Changes in the sport community after removing the communication range restriction.

From the previous discussion, we can see that any device in the DCIM can build a CIR with other devices only if they are in its communication range. For experimental purposes, we try to change this restriction to have a global communication range using networking protocols such as UPnP technology, which allows devices to discover each other based on their common network provider. To apply this in the airport scenario, we assume that any object entering the airport has the ability to interact and build CIRs with any other object in the same airport assuming they are sharing the airport WiFi. We discovered that, in this case, a device can join more communities and interact with more devices even if they are outside of its range. However, whenever the community is formed, it will not change while the object is moving unless the object leaves the airport. Taking the same example of Figure 10, but with range restriction removed, we can see the stability in the sport community (as shown in Figure 13) after we stopped adding more travelers, unlike the dynamic result achieved with the range constraint.

Overlooking the owner’s rules key-values, we can observe that when leaving the object without any rules, it will be able to join any community of interest that matches their profile, which may cause overload in some devices and thereby dissatisfy the owners.

### 5.3. DCIM and Clustering Techniques

We can observe from the DCIM that a kind of clustering technique is used with the aim to increase interactivity between the community or clustered mobile members, increase scalability of IoT applications, and allow overlapping between clusters. Similar to some available clustering techniques, the proposed model follows specific processes to identify and distribute members among communities.

## 6. Conclusions and Future Work

In this research, we focused on analyzing the integration of the social aspects in IoT systems. To do so, we identified the capabilities and properties of different IoT objects that enable them to socialize and become involved with other objects. In addition, we identified the relationships and proposed a new relationship that can be possessed by IoT objects to allow mimicking of social behavior. Finally, a Dynamic Community of Interest Model was proposed and implemented using Agent-Based Modeling through the NetLogo simulation and modeling platform. The proposed model exploits common interests of objects, communication range, and social interaction rules to dynamically form communities of IoT objects. Simulation results show that the model is scalable in terms of the number of objects allowed to be in a community and the number of communities that can be formed at the same time. The communities formed dynamically allow an object to interact with other objects based on a shared interest. Collaborating with interested objects in a community leads to better efficiency, fast service, and more effective interaction in a dynamic mobile IoT environment.

The beneficiaries of the proposed model are those companies, individuals, or entities who desire social IoT systems in smart cities. As future work, we plan to develop a trustworthiness management system [36] and integrate it within the DCIM model in the Application layer. We also plan to implement the DCIM approach in several real-life scenarios with a sizable number of IoT entities.

## Figures and Tables

**Table 1:** Main differences between Dynamic Community of Interest Model (DCIM) and other implementations.

Reference	Object Relationships	IoT Devices Considered	Community Formation	Technique Used for Community Formation	Architecture Proposed	Architectural Distribution	Object Relationships Implementation Status
**Atzori** [4,5]	POR, CLOR, CWOR, OOR, SOR.	IoT objects	Friendship-based	Object relationship based	SIoT Architecture, Client/server Architecture	Centralized	SWIM mobility simulator
**Kosmatos** [7]	General	RFID, smart objects	Possible	Social Networks (SN), Blogject Community	IoT middleware architecture	Centralized	Conceptual level (not implemented)
**Farris** [9]	POR, CLOR, CWOR, OOR, SOR.	Social virtual objects	-	-	Cloud-based SIoT architecture	Distributed cloud solution	(Lysis) Cloud-based platform, and use case scenario
**Nitti** [10]	POR, CLOR, CWOR, OOR, SOR.	IoT objects	Possible	Link selection strategy (Friend list, FOAF)	-	-	Simulation
**Yue** [21]	General	Sensors, RFIDs, smart phones	Community of interest	Operation based through DataClouds	Community-based architecture	Centralized	Simulation
**Misra** [22]	General	Basic nodes, IoT nodes	Mutual friends’ community	Graph mining approach	-	-	Not implemented
**Girau** [13]	Rule based	IoT object	Community of interest	Client-side, Server-side, and Hybrid solution for groups’ management.	-	-	Experimental platform through web based simulation
**An** [14]	General	Mobile nodes	Cohesive subgroups	Nodes social relations cognition algorithm	-	-	Not implemented
**Ding** [15]	General	Information, objects, people	-	-	Clustering SN, Internet, IoT	-	Not implemented
**Nitti** [17]	POR, CWOR, SOR.	Vehicle, RSUs	-	-	-	-	SUMO simulator
**Alam** [18]	POR, CWOR, SOR, GOR	Vehicle, RSUs, HBUs	-	-	Cyber-physical architecture for SIoV	Distributed	SUMO simulator
**Mäkitalo** [23]	Predefined based on object role	Human, mobile devices	-	-	SDP architecture	Centralized	Prototype implementation for (SDP)
**Proposed approach**	Common Interest relationship	Heterogeneous Objects	Community of interest, DCIM	Clustering-rules based technique	SIoT architecture	Distributed	NetLogo simulator
